# Gaps in Team Communication About Service Statistics Among Health Extension Workers in Ethiopia: Secondary Data Analysis

**DOI:** 10.2196/20848

**Published:** 2020-09-08

**Authors:** Seohyun Lee, Eunji Kim, Tekaligne Birhane Desta

**Affiliations:** 1 Yonsei University Wonju Republic of Korea

**Keywords:** team communication, health extension workers, mobile communication, mobile data collection, Ethiopia, health worker, communication, data

## Abstract

**Background:**

In Ethiopia, health extension workers (HEWs) are deployed across the country by the government to meet public health needs. Team communication is important for effective teamwork, but community health workers in low-resource settings like Ethiopia may face challenges in carrying out team meetings to compile service statistics. This is due to the nature of their outreach activities, which requires extensive travel.

**Objective:**

This study aimed to identify gaps in team communication about service statistics among HEWs in Ethiopia. Considering mobile communication and data collection as tools for bridging these gaps, we examined disparities in access to electricity, which has been identified as one of the major barriers to this approach.

**Methods:**

Data from the most recent Performance Monitoring and Accountability 2020 service delivery point survey were used for our analysis. Logistic regression analysis was performed to identify disparities in team communication on service statistics for family planning, which is a major component of the HEW’s job. Disparities were examined across health facilities with different levels of HEW integration in their staffing structure (ie, no HEWs, at least one HEW, or only HEWs). Additionally, a chi-square test was conducted to examine disparities in access to electricity to explore the potential of mobile communication and data collection integration.

**Results:**

In total, 427 health facilities of four different types (ie, hospitals, health centers, health posts, and health clinics) were included in our analysis. At most health posts (84/95, 88%), only HEWs were employed; none of the health clinics integrated the HEW model into their staffing structure. Among the 84 health posts, the odds of having team meetings on family planning service statistics in the past 12 months were 0.48 times the odds of those without HEWs (*P*=.02). No statistically significant differences were found between HEW-only facilities and facilities with at least one HEW. Most health facilities (69/83, 83.13%) with HEWs as the only staff had no electricity at the time of the survey while 71.25% (57/80) had intermittent access (ie, service disruption lasting 2 or more hours that day). There were statistically significant differences in electricity access among health facilities with different levels of HEW integration (*P*<.001).

**Conclusions:**

Facilities employing only HEWs were less likely to have regular team meetings to discuss service statistics. Since their responsibilities involve extensive outreach activities, travel, and paper-based recordkeeping, empowering HEWs with mobile communication and data collection can be a workable solution. The empirical evidence regarding disparities in electricity access also supports the need for and the feasibility of this approach.

## Introduction

Team-based interactions among health workers have been considered to be drivers for productivity, effective service delivery, cooperation, and quality improvement [1,2]. When teams communicate on a regular basis, health workers can share workload, make more informed decisions, and exchange new ideas in a more efficient manner [3]. In resource-limited settings, building teamwork through communication can be challenging but critical. In fact, previous studies have demonstrated that communication is one of the key factors for establishing teamwork in low- and middle-income countries (LMICs). A study conducted in Tanzania identified team communication as one of the main strategies for team implementation efforts in improving postpartum care [4]. Another study that reviewed 47 studies regarding teamwork among community health workers (CHWs) and health care teams found regular team meetings with specific purposes to be an essential component of the team’s interdependence [2]. Another study from India revealed that team communication for cross-checking records, planning outreach sessions, or cross-training is an important part of practices managed by CHWs [5]. In the Zambian context, Yeboah-Antwi et al [3] identified good communication and sharing information as two core factors that characterize teamwork.

In LMICs where a shortage of skilled health professionals triggered the deployment of CHWs, team communication can be even more challenging. Specifically, scheduling a regular team meeting may not be feasible since the majority of the CHW’s work involves community outreach that requires extensive travel. CHWs are in a unique position where they function as a liaison between the community and the health system. According to the World Health Organization (WHO) guidelines, the tasks of CHWs defined by International Labour Organization include providing education and advice for communities, conducting outreach efforts, and managing medical supplies [6]. In other words, CHWs are responsible for supporting communities as well as the health system, requiring frequent travel and activities outside of health facilities.

Ethiopia is one of the low-income countries that implemented a community-based health program involving CHWs called health extension workers (HEWs). In 2003, the Ethiopian Federal Ministry of Health introduced the Health Extension Program, and as part of that program, HEWs have been recruited, trained, and salaried by the government to meet the public health needs of the community [7-9]. HEWs are mostly female with at least a 10th-grade education and are from the communities they serve, allowing them to build trust and rapport with the community [8]. According to task analysis studies for HEWs, they spend a majority of their time on travel or outreach activities, which may limit the possibilities for effective team communication. Studies have noted that HEWs spend approximately 75% of their working time performing outreach activities [8,10,11]. A time and motion study found that they spend 15.5% of their time traveling between work activities [12]. As a result, gathering for a regular staff meeting at the same location may not be a workable solution for HEWs to share information or ideas.

Indeed, the HEW’s difficulty due to extensive travel lies not only in the flow of information per se but also in the quality of information shared among HEWs and their supervisors [13]. Previous literature suggested that the existing paper-based data collected between travels by HEWs pose a challenge in terms of poor quality and extra burden for aggregation or reporting [14,15]. Studies have pointed out that recordkeeping and reporting of service data is one of the main tasks assigned to HEWs, requiring them to bring a paper-based field book while traveling for outreach activities [14,16]. After returning to health facilities, HEWs need to spend extra time on compiling or validating the data and generating service statistics for reporting. Working with paper-based field book can be inefficient, and there is a risk for loss of data or limited validity.

In this context, the primary objective of this study is to identify gaps in team communication on service statistics among health facilities with no HEWs, at least one HEW, or exclusively HEWs comprising their staffing structure. Based on previous literature on the impact of mobile technology on teamwork and communication, we considered mobile communication and data collection as good candidates for bridging these gaps [17]. To identify potential challenges in mobile communication and data collection, we examined disparities in access to electricity across different health facilities. In fact, evidence from a systematic review on health workers’ use of mobile devices in low-resource settings found that poor access to electricity is one of the main barriers to mobile communication and data collection [18].

## Methods

### Data and Scope

For analysis, the most recent Performance Monitoring and Accountability 2020 (PMA2020) service delivery point data were used. PMA2020 is an initiative for improving family planning in LMICs supported by the Bill and Melinda Gates Foundation and collects a nationally representative sample data through mobile data collection [19]. Specifically, the PMA2020 Ethiopia Round 6 Service Delivery Point 2018 data were used for the study. PMA2020 service delivery point data comprise a nationally representative data set collected at the health facility level and is publicly available upon request [19]. For data collection, trained resident enumerators visit the sampled facilities and conduct surveys via mobile phones [20]. By utilizing mobile data collection strategy for large-scale surveys on various health issues such as family planning or water and sanitation, PMA2020 provides rapid and high-quality data for decision making and policy design.

Ethiopia Round 6 data contain information about 476 health facilities and their service provision and quality, with special attention paid to family planning. In total, the 427 health facilities that reported valid responses to the key questions needed for our analysis were included in this study. For example, facilities that did not provide information about the number of each staff position were excluded since disparities were compared across health facilities with different levels of HEW integration in the staffing structure. Health facilities were categorized as those with no HEWs, at least one HEW, or HEWs as the only staff position. In the context of Ethiopia’s health system, two HEWs are usually deployed to each health post to cover 3000 to 5000 people to improve primary health care [16]. In addition to health posts, three other types of health facilities were included for analysis—hospitals, health centers, and health clinics. Although data on pharmacies, retail outlets, and others were available, they were not included in the analysis because their focus is not on the provision of health services.

### Measurements

The primary outcome measure is the binary variable of team communication on service statistics for family planning, which is the main responsibility of the HEW [9,16,21]. It is defined as having any meetings with staff to discuss service statistics or inventory for family planning in the past 12 months [19]. Examples of family planning service statistics include the total number of visits or the number of emergency contraception units sold in the past completed month [19]. Explanatory variables include the level of integration of HEWs into the staffing structure, location (ie, urban or rural areas), knowledge of the catchment population size, and recent external supervisor visit and support from nongovernmental organizations (NGOs) for family planning services. The level of integration of HEWs into the staffing structure was measured categorically—facilities with no HEWs, with at least one HEW, or comprising entirely of HEWs. Recent external supervisor visits were categorized as having occurred more than 6 months ago versus within the past 6 months. Support from NGOs for family planning services included funding or other types of support in the past 12 months, like training, technical assistance, or supplies [19]. The reasoning behind adding this variable comes from previous studies that discussed NGOs’ special focus on management strategies and relatively higher level of technical competence than government or private facilities for the implementation of family planning services in Ethiopia [22,23].

Having access to electricity can facilitate wireless communication, charging mobile devices, and maintaining other electronic devices for communication and data management. To assess the disparities in access to electricity as the potential barrier to mobile communication and data collection, two measures related to electricity access were examined. The first measure looked at whether the facility had electricity at the time of the survey, and the second measure investigated whether there was intermittent access at the facility (ie, service disruption of 2 or more hours on the day the survey was conducted).

### Statistical Analysis

Descriptive statistics were used to provide an overview of the characteristics of health facilities that have no HEWs, at least one HEW, or comprised only HEWs. To study the association between team meetings on service statistics and the integration of HEWs into the staffing structure, we conducted a binary logistic regression analysis. Predicted probabilities for having team communication in the past 12 months among health facilities were estimated and presented graphically. A chi-square test was performed to assess disparities in access to electricity among health facilities with no HEWs, at least one HEW, or only HEWs. Stata (version 15, StataCorp LLC) was used for statistical analysis [24].

## Results

### Descriptive Statistics

Descriptive statistics are presented in Table 1. In terms of facilities, four types representing the health system of Ethiopia—hospitals, health centers, health posts, and health clinics—were identified. The hierarchy of the primary health care unit in Ethiopia is as follows: the *woreda* (district) health office oversees health centers, which in turn supervise five health posts [16,25]. Additionally, hospitals and health clinics provide support for the health system. In total, 84 out of 95 health posts (88%) comprised only HEWs; no other facility type had this composition. There was at least one HEW deployed at all health posts. On the other hand, health clinics in the sample did not integrate HEWs at all into their staffing structure. Among facilities that had no HEWs, 45.1% (n=105) were hospitals, 45.9% (n=107) were health centers, and 9% (n=21) were health clinics.

**Table 1:** Descriptive statistics for health facilities (source: PMA2020 Ethiopia Round 6 Service Delivery Point 2018 [19]).

Variable			Staffing structure, n (%)				
			No HEWs^a^ (n=233)		At least one HEW (n=110)		Only HEWs (n=84)
**Facility type**							
	Hospital	105 (45.1)		3 (2.7)		0 (0)	
	Health center	107 (45.9)		96 (87.3)		0 (0)	
	Health post	0 (0)		11 (10)		84 (100)	
	Health clinic	21 (9.0)		0 (0)		0 (0)	
**Location**							
	Rural	122 (52.4)		41 (37.3)		73 (86.9)	
	Urban	111 (47.6)		69 (62.7)		11 (13.1)	
**Managing authority**							
	Government	210 (90.1)		110 (100)		84 (100)	
	Private	22 (9.4)		0 (0)		0 (0)	
	Other	1 (0.4)		0 (0)		0 (0)	
**Knowledge of catchment population size**							
	Not applicable (no catchment area)	17 (7.3)		4 (3.6)		1 (1.2)	
	No	18 (7.7)		5 (4.6)		1 (1.2)	
	Yes	198 (85.0)		101 (91.8)		82 (97.6)	
**Recent external supervisor visit**							
	More than 6 months ago	24 (10.3)		21 (19.1)		6 (7.1)	
	Within the past 6 months	209 (89.7)		89 (80.9)		78 (92.9)	
**Received support in the past 12 months from an NGO^b^ for family planning services**							
	No	101 (43.4)		26 (23.6)		50 (59.5)	
	Yes	132 (56.7)		84 (76.4)		34 (40.5)	
**Had meetings with staff to discuss services statistics for family planning in the past 12 months**							
	No	45 (19.3)		12 (10.9)		24 (28.6)	
	Yes	188 (80.7)		98 (89.1)		60 (71.4)	

^a^HEW: health extension worker.

^b^NGO: nongovernmental organization.

With regard to location, 86.9% (n=73) of the facilities with only HEWs were located in rural areas, whereas 62.7% (n=69) of those with at least one HEW were located in urban areas. Among health facilities with no HEWs, 52.4% (n=122) were located in rural areas and 47.6% (n=111) were in urban areas. All facilities with at least one HEW or HEWs as the only staff were managed by the government. This result reflects the fact that HEWs are employed, trained, and salaried by the government [10,26].

When asked about the size of the catchment population, facilities with HEWs as the only position had the highest proportion of positive answers (n=82, 97.6%). For facilities with no HEWs or at least one HEW, more than 5% (n=35 and 9, respectively) answered that they had no catchment area or did not know the size of the catchment population.

In terms of recent external supervisor visits, facilities with HEWs as the only staff had the highest proportion of having a supervisor visit within the past 6 months (n=78, 92.9%). For facilities with no HEWs or at least one HEW, it was 89.7% (n=209) and 80.9% (n=89), respectively.

As for support from NGO for family planning services in the last 12 months, facilities with at least one HEW had the highest proportion of having received this support (n=84,76.4%); 56.7% (n=132) of facilities with no HEWs and 40.5% (n=34) of those with HEWs as the only staff position reported that they had received support from NGOs.

When comparing experiences of team communication among health facilities, those comprising only HEWs had the lowest proportion of team meetings about service statistics in the past 12 months (n=60, 71.4%). Overall, 80.7% (n=188) of facilities with no HEWs and 89.1% (n=98) of those with at least one HEW had meetings to discuss service statistics for family planning in the past 12 months.

### Disparities in Team Meetings About Service Statistics

**Figure 1:**
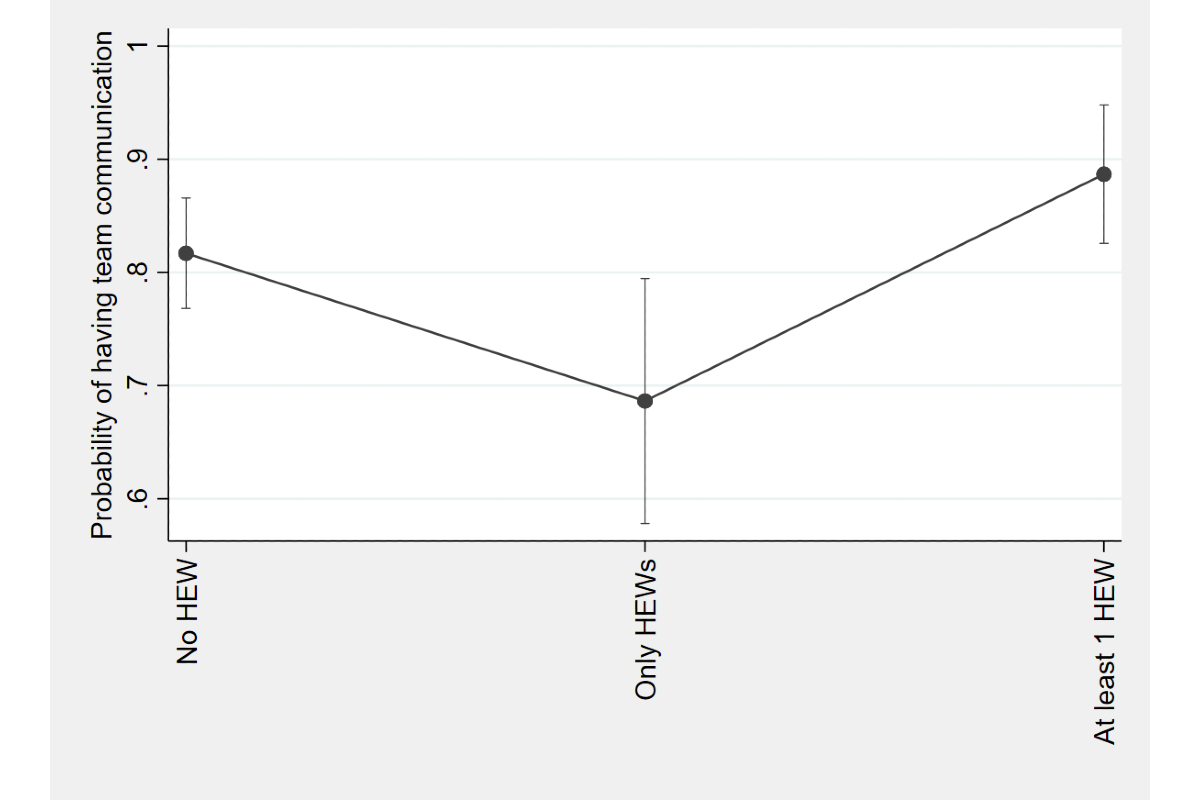
Predicted probabilities of having team communication in the past 12 months among facilities with different levels of health extension worker (HEW) integration.

Table 2 shows the results from the logistic regression analysis that examined the association between team communication on family planning service statistics and the representation of HEWs in staffing structures. For health facilities with HEWs as the only staff, the odds of having team meetings on family planning service statistics during the last 12 months were 0.48 times compared to those without HEWs (*P*=.02). However, there was no statistically significant difference between HEW-only facilities and facilities with at least one HEW plus other staff. Figure 1 presents the estimated predicted probabilities of having team communication among facilities with different levels of HEW integration in their staffing structure.

The association between knowledge on catchment population size and team communication experience was as follows: facilities that were aware of the size of the catchment area were more likely to report instances of team communication regarding service statistics in the last 12 months compared to those that did not have a catchment area or had no knowledge of its size (odds ratio [OR] 2.21, *P*=.03).

**Table 2:** Logistic regression model for the association between team communication on family planning service statistics and the integration of health extension workers (HEWs) into the staffing structure (source: PMA2020 Ethiopia Round 6 Service Delivery Point 2018 [19]).

Variable		Team communication in the past 12 months		
		Odds ratio (95% CI)	SE	*P* value
**Integration of HEWs into staffing structure (ref^a^: no HEWs)**				
	At least one HEW	1.78 (0.88-3.62)	0.65	.11
	Only HEWs	0.48 (0.25-0.90)	0.15	.02
**Location (ref: rural)**				
	Urban	0.68 (0.39-1.18)	0.19	.17
**Knowledge of catchment population size (ref: none or no catchment area)**				
	Yes	2.21 (1.06-4.64)	0.84	.03
**Recent external supervisor visit (ref: >6 months ago)**				
	Within the past 6 months	1.02 (0.46-2.25)	0.41	.96
**Received support in the last 12 months from an NGO^b^ for family planning services (ref: no)**				
	Yes	1.47 (0.88-2.45)	0.39	.14

^a^ref: reference.

^b^NGO: nongovernmental organization.

### Disparities in Access to Electricity

Table 3 illustrates the disparities in access to electricity among health facilities with no HEWs, at least one HEW, and only HEWs in their staffing structure. When asked if they had electricity at the time of the survey, the majority of the respondents from facilities with no HEWs (190/233, 81.55%) and those with at least one HEW (88/110, 80.0%) said that they had access to electricity. On the contrary, 83.13% (69/83) of the respondents from health facilities with only HEWs responded that they had no electricity at the time of the survey. Chi-square test results indicate that there was a statistically significant difference in this regard (*P*<.001).

**Table 3:** Disparities in access to electricity for health facilities with different levels of health extension worker (HEW) integration into their staffing structure.

Variable		Access to electricity		*P* value^a^
		No, n (%)	Yes, n (%)	
**Intermittent electricity access (ie, disruption lasting 2 or more hours)**				.001
	No HEWs	156 (66.95)	77 (33.05)	
	At least one HEW	70 (64.22)	39 (35.78)	
	Only HEWs	23 (28.75)	57 (71.25)	
**Has electricity at this time**				.001
	No HEWs	43 (18.45)	190 (81.55)	
	At least one HEW	22 (20.00)	88 (80.00)	
	Only HEWs	69 (83.13)	14 (16.87)	

^a^Chi-square test.

However, when the respondents were asked if they had electricity at least on the day of the survey, but with a service disruption of 2 or more hours, 71.25% (n=57) of facilities with only HEWs in their staffing structure said “yes.” For facilities with no HEWs and those with at least one HEW, the percentages were much lower: 33.05% (n=77) and 35.78% (n=39), respectively. Again, the chi-square test result indicated that there was a statistically significant difference in intermittent access to electricity among health facilities with different levels of HEW integration in their staffing structure (*P*<.001).

## Discussion

### Principal Findings

This study demonstrated that health facilities in Ethiopia with only HEWs are less likely to have team meetings to discuss service statistics and more likely to experience unstable access to electricity than those without HEWs. To further explore the gaps in team communication, we focused on possible challenges among HEWs in terms of scheduling a meeting in a physical location and generating basic statistics from the paper-based field books they carry while traveling.

First of all, convening a meeting at a physical location may not be feasible for HEWs who travel a lot. Given the HEWs’ role as CHWs, the majority of their working time is devoted to outreach activities and extensive travel [12]. As evidenced by previous studies, they spend approximately 75% of their working time on outreach activities and 15.5% on travel [8,10-12]. We confirmed with one health bureau staff that, in general, 80% of the HEW’s working time or 4 days per week is spent on visiting households or conducting outreach activities. Consequently, HEWs may face difficulties in meeting team members to discuss service statistics, and the results from this study provide evidence on gaps between facilities with only HEWs and those without HEWs.

Another important aspect of these gaps in team communication on service statistics is the extra burden involved to generate basic statistics. As previous studies have stated, part of the reason for these gaps may come from the inefficiencies associated with the paper-based recordkeeping practice among HEWs [13,27]. When HEWs travel for their outreach activities, they usually carry a paper-based field book for recording purposes. In this field book, they record information about clients as well as services provided. After they return from the community, HEWs spend extra hours updating various information sheets for reporting, such as tally sheets, kebele (lowest administrative unit) profiling templates, or follow-up cards for various types of health conditions [8]. In addition to the inefficiencies associated with paper-based records, there is a risk for loss, duplication, or invalidity of data. These challenges, which are related to service statistics, can explain the gaps identified in this study to some extent.

Building upon previous studies, we considered mobile communication and data collection as a potential solution for these challenges [17]. In doing so, we identified disparities in access to electricity, which are potential barriers to mobile communication and data collection adoption. The disparities in access to electricity identified from this study support the need for and the feasibility of this approach. According to our results, health facilities with HEWs as the only staff had a substantially lower level of access to electricity at the time of the survey (16.87%) compared to facilities with no HEWs (81.55%) or with at least one HEW (80.0%). However, it was noted that 71.25% of facilities with only HEWs had intermittent access to electricity, implying the possibility of using it for charging mobile devices between travels.

Ideally, stable access to electricity and wireless networks would be needed for the effective use of mobile devices among HEWs. Currently, there is a pilot program involving an electronic community health information system for some health posts in the Oromia region so that HEWs can use a tablet computer for recordkeeping purposes. In addition, previous studies on HEWs in Ethiopia have already examined the potential for and the feasibility of using mobile devices for communication and data collection [13,28-31]. A qualitative study based on the Ethiopian context suggested that HEWs who participated in the intervention involving smartphones for data collection saw it as a “helping hand” [31].

Our study has limitations in terms of generalizability and the variables available from the secondary data. First, the scope of the analysis is focused on HEWs in Ethiopia although CHWs are deployed across many LMICs. The rationale for choosing Ethiopia as a target for this exploratory analysis was based on its well-structured staffing model for primary health care at the community level involving CHWs employed by the government. Future studies can explore the gaps in team communication on service statistics among CHWs in other contexts.

Secondly, the analysis is limited to the available variables from the PMA2020 data, which is secondary data collected by a global initiative. Most importantly, team communication for sharing information on service statistics can be assessed by various measures but the PMA2020 data provided only information on whether the facility had staff meetings on service statistics for family planning for the past 12 months or not. Also, additional measures that can examine facilitators and barriers for mobile communication and data collection should be examined in addition to electricity access. Based on our findings, future studies can investigate other variables that can explain the gaps in team communication on service statistics and the potential factors for assessing the feasibility of mobile communication and data collection.

As an exploratory analysis, this study attempted to provide empirical evidence on gaps in team communication about service statistics among CHWs in Ethiopia and explore the potential for mobile communication and data collection. As evidenced by current pilot programs and previous literature, utilization of mobile communication and data collection by HEWs can help bridge the gaps in team communication concerning service statistics.

### Conclusions

This study aimed to examine the gaps in team communication for discussing service statistics among HEWs in Ethiopia. On the one hand, HEWs face challenges in convening for meetings at a physical location due to the extensive outreach activities and travel associated with their line of work. On the other hand, generating basic statistics from the paper-based field books that HEWs carry while traveling requires extra effort. In this context, we explored the potential for mobile communication and data collection and discussed how it can transform the way that HEWs cross-check and share service statistics data. Building upon the findings from this study, the workflow of HEWs can be improved for efficient team communication on service statistics.

## Abbreviations

CHW: community health worker
HEW: health extension worker
LMIC: low- and middle-income country
NGO: nongovernmental organization
OR: odds ratio
PMA2020: Performance Monitoring and Accountability 2020
WHO: World Health Organization
